# Perspective: Collagen induced platelet activation *via* the GPVI receptor as a primary target of colchicine in cardiovascular disease

**DOI:** 10.3389/fcvm.2022.1104744

**Published:** 2023-01-19

**Authors:** Gabrielle J. Pennings, Caroline J. Reddel, Vivien M. Chen, Sonali R. Gnanenthiran, Leonard Kritharides

**Affiliations:** ^1^Vascular Biology Group, ANZAC Research Institute, The University of Sydney, Concord, NSW, Australia; ^2^Department of Cardiology, Concord Repatriation General Hospital, Concord, NSW, Australia; ^3^Department of Haematology, Concord Repatriation General Hospital, Concord, NSW, Australia; ^4^Platelet, Thrombosis Research Laboratory, ANZAC Research Institute, The University of Sydney, Concord, NSW, Australia; ^5^The George Institute for Global Health, University of New South Wales, Newtown, NSW, Australia

**Keywords:** colchicine, platelet activation, GPVI, cardiovascular disease (CVD), stroke

## Abstract

Colchicine has been demonstrated to reduce cardiovascular death, myocardial infarction (MI), ischemic stroke, and ischemia-driven coronary revascularization in people with coronary artery disease (CAD). These reductions were observed even in patients already taking antiplatelet therapy. As well as having anti-inflammatory effects, colchicine demonstrates antiplatelet effects. We propose that colchicine's antiplatelet effects primarily target collagen-induced platelet activation *via* the collagen receptor, glycoprotein (GP)VI, which is critical for arterial thrombosis formation. In settings such as stroke and MI, GPVI signaling is upregulated. We have demonstrated *in vitro* that therapeutic concentrations of colchicine lead to a decrease in collagen-induced platelet aggregation and alter GPVI signaling. Clinical studies of colchicine given for 6 months lead to a significant reduction in serum GPVI levels in CAD patients, which may ameliorate thrombotic risk. Future evaluation of the effects of colchicine in clinical trials should include assessment of its effects on collagen-mediated platelet activation, and consideration be given to quantifying the contribution of such antiplatelet effects additional to the known anti-inflammatory effects of colchicine.

## Introduction

Cardiovascular diseases (CVD), including coronary artery disease (CAD) and cerebrovascular disease, remain the main cause of death in the world (1). The contribution of platelets to CVD initiation and progression is well evidenced and has now extended beyond thrombosis alone to include a role in inflammation [reviewed in Gawaz et al. (2), Nording et al. (3), and Rondina et al. (4)]. Platelets respond to both sub-endothelial collagen (upon endothelial dysfunction/disruption) *via* the collagen receptors [integrin α_2_β_1_ and glycoprotein (GP)VI], and to thrombin (generated at the site by activation of the coagulation system) *via* the protease-activated receptors (PAR)-1 and -4. The platelet activation response occurs rapidly and includes shape change, release of granular contents from both alpha- and dense-granules, and conformational change of receptors such as GPIIb/IIIa which allows for platelet aggregation. The dense granule release leads to secretion of adenosine diphosphate (ADP) which is involved in the secondary wave of platelet activation which enhances the platelet activation response.

Antiplatelet therapy in CVD [reviewed in Behan and Storey (5) and Passacquale et al. (6)] began with inhibition of the cyclo-oxygenase (COX)-1 pathway by aspirin, followed by inhibition of the ADP receptor, P2Y_12_, as an alternative or adjunct therapy to aspirin, and GPIIb/IIIa inhibition in acute coronary syndromes. In all cases, more potent or extensive antiplatelet inhibition has been associated with increased risk of bleeding. Completed (7, 8) and ongoing trials include alternative antiplatelet targets, including a collagen receptor (GPVI) (9, 10), and the thrombin receptors (PAR-1 and−4) [reviewed in Abdulsattar et al. (11) and Li et al. (12)]. Anti-inflammatory targets, including interleukin (IL)-1β and nucleotide-binding domain leucine rich-repeat containing protein (NLRP3) (13–18) have also been recently explored. Recent studies have identified that colchicine may work as both an anti-inflammatory [reviewed in Martinez et al. (19)] and antiplatelet agent (20, 21).

In this perspective, we aim to highlight the need to evaluate collagen-mediated platelet activation in clinical environments and in cardiovascular trials. This may clarify the potential role of this under-investigated activation pathway in mediating the effects of colchicine as an adjunctive CVD therapeutic agent.

## Platelet function studies and CAD clinical trials

COX-1 and P2Y_12_ inhibition have been the cornerstones of antiplatelet therapy for cardiovascular disease for several decades (6, 22, 23). While dual antiplatelet therapy (e.g., aspirin and clopidogrel) has proven to be effective in reducing cardiovascular events in unstable angina pectoris patients and post PCI (24, 25), this treatment has some limitations such as residual high on-treatment platelet reactivity to ADP (26) and the bleeding complications caused by more potent P2Y_12_ inhibitors (27). Importantly, trials adjusting therapy based on testing platelet function using the level of P2Y_12_ inhibition were not effective in improving cardiovascular outcomes (28–30), suggesting limitations to our conventional approaches of assessing platelet function in patients with CVD. P2Y_12_ inhibition can have additional unexpected effects on platelet reactivity to both collagen and thrombin which are not explored during conventional platelet testing (31). While conventional platelet function testing is not currently recommended in patients undergoing PCI (32), there has been some success in ACS with de-escalation based on platelet function testing (33).

Despite the availability of diverse platelet function assays (34), clinical trials have focused on assays such as aggregometry, and ADP-dependent point of care assays (VerifyNow and TEG) and vasodilator-stimulated phospho-protein (VASP) phosphorylation [reviewed in Fontana et al. (35)]. Aggregation assays are dependent on both the agonists and the concentrations used, such as stimulating the P2Y_12_ receptor (29, 33). Additionally, results can differ according to sample type. Light transmission aggregometry (LTA) requires the use of platelet rich plasma (PRP), whereas impedance aggregometry allows for the use of either PRP or whole blood, and the latter may permit leukocyte contributions to platelet activation which may be more physiological. VASP phosphorylation, while informative for activity/signaling or response of the P2Y_12_ receptor to stimulation by ADP and thus the secondary augmentation response, does not inform in relation to other pathways of platelet activation. Despite platelets being anucleate and relatively small, their signaling and activation pathways are both complex and interconnected. The concept of individualized therapy is receiving more attention (29, 33), and rather than only looking at drug responsiveness with one assay/agonist, e.g., ADP, inclusion of other agonists such as collagen and thrombin may be informative.

## Collagen-mediated platelet activation and the GPVI receptor

Platelet activation through the collagen receptors (integrin α_2_β_1_ and GPVI) is critical for the formation of arterial thrombosis and can lead to blockage of vessels resulting in myocardial infarction (MI) or stroke. Whereas, inhibiting more traditional targets such as GPIIb/IIIa (aka integrin αIIbβ_3_), P2Y_12_, and thromboxane A_2_ can lead to bleeding complications, it is possible that novel receptor targets such as the collegen receptor will not cause bleeding (9, 10, 36).

GPVI is solely expressed on platelets, as a mixture of monomers and dimers (37). It is the primary receptor for collagen, and collagen-mediated platelet activation *via* GPVI is dependent on receptor density (38). Binding between collagen and GPVI enhances activation of integrins such as α_2_β_1_ which increases adhesiveness of the platelets (39, 40). In the absence of GPVI, α_2_β_1_ is required for collagen-mediated platelet activation (40) but under physiological conditions GPVI is the main collagen receptor. Interestingly, collagen stimulation (at low concentrations) *via* GPVI can lead to secretion of platelet granule contents without causing other overt activation (expression of P-selectin (CD62P) or GPIIb/IIIa conformational change), aggregation or shape change (41). Thus, strategies leading to partial inhibition of GPVI signaling are attractive in CVD due to the ability to target pathological granule secretion while leaving the major haemostatic pathways intact.

Surface levels of GPVI expression are increased in acute coronary syndromes (ACS) independent of traditional markers of MI [e.g., creatine kinase (CK) and troponin T] (42). Moreover, patients who had increased GPVI levels also had increased platelet aggregability post coronary stenting despite dual antiplatelet therapy (42). GPVI expression levels could also be a potential marker of MI risk prior to the event (42–44). Additionally, ACS patients have an elevated number of GPVI receptors on circulating platelets before (Day 0) and after stenting (Day 1) when compared to patients with stable angina pectoris (SAP) which lasts up to 2–4 days post-PCI, and patients with an elevated level of GPVI at admission tended to have a poorer clinical prognosis (42). Enhanced collagen-induced aggregation in the presence of COX-1 inhibition (aspirin) can be indicative of future ACS events in otherwise healthy individuals who have a known family risk of early-onset CAD–only those individuals whose collagen-induced aggregation remained elevated after 2 weeks of aspirin, tested in whole blood, had an ACS event during follow-up, further underscoring the potential importance of collagen-mediated platelet activation in ACS (45).

During platelet activation, in response to both soluble platelet agonists and shear, GPVI forms dimers that preferentially bind to fibrous collagen when compared to the GPVI-monomer (46, 47). Inhibition of the binding between the GPVI-dimer and immobilized collagen leads to decreased platelet adhesion and aggregation (48). GPVI-dimer expression levels, but not total GPVI levels, have also been demonstrated to be elevated in stroke, with elevation being noted for ≥90 days post-stroke (49). This increase was associated with increased platelet activation and with stroke severity. Cleavage of GPVI by metalloproteinases is a feature of collagen-mediated platelet activation (50). This generates soluble GPVI (sGPVI), and increased levels of sGPVI have been demonstrated in stroke (51).

Revacept, a GPVI-Fc fusion protein, has shown some promise in reducing cerebral infarct volume and improving functional outcomes in a stroke model without bleeding complications (36). Revacept in combination with guideline recommended antiplatelet therapy has now undergone phase II clinical trials in symptomatic carotid stenosis. The clinical trial (*n* = 158 patients randomized to three groups; placebo, 40 and 120 mg Revacept) assessed the safety and efficacy of the treatment with the following end points—any cerebrovascular events (including stroke or MI) or bleeding complications. A 54% risk reduction in the safety and efficacy end points was observed in the highest dose (120 mg Revacept) group, demonstrating the potential of GPVI as a therapeutic target and a clear indication of the importance of collagen-mediated platelet activation (9).

## Colchicine inhibits collagen mediated platelet activation

While it is well-known that colchicine affects the typical inflammatory cells such as monocytes and neutrophils, it is becoming increasingly evident that colchicine also influences platelets. We have recently reviewed the effect of colchicine on platelet function in both *in vitro* and *in vivo* settings (52).

Studies examining platelet aggregation in response to *in vitro* colchicine show that whereas inhibition of aggregation to several common platelet agonists requires micromolar concentrations of colchicine, nanomolar concentrations inhibit certain responses to thrombin (53, 54) and calcium ionophore (55). In our studies (20), platelet aggregation in whole blood and in platelet rich plasma in response to collagen (but not ADP) were inhibited by nanomolar concentrations of colchicine indicating that colchicine may be a novel method of biased targeting of GPVI.

**Figure 1:**
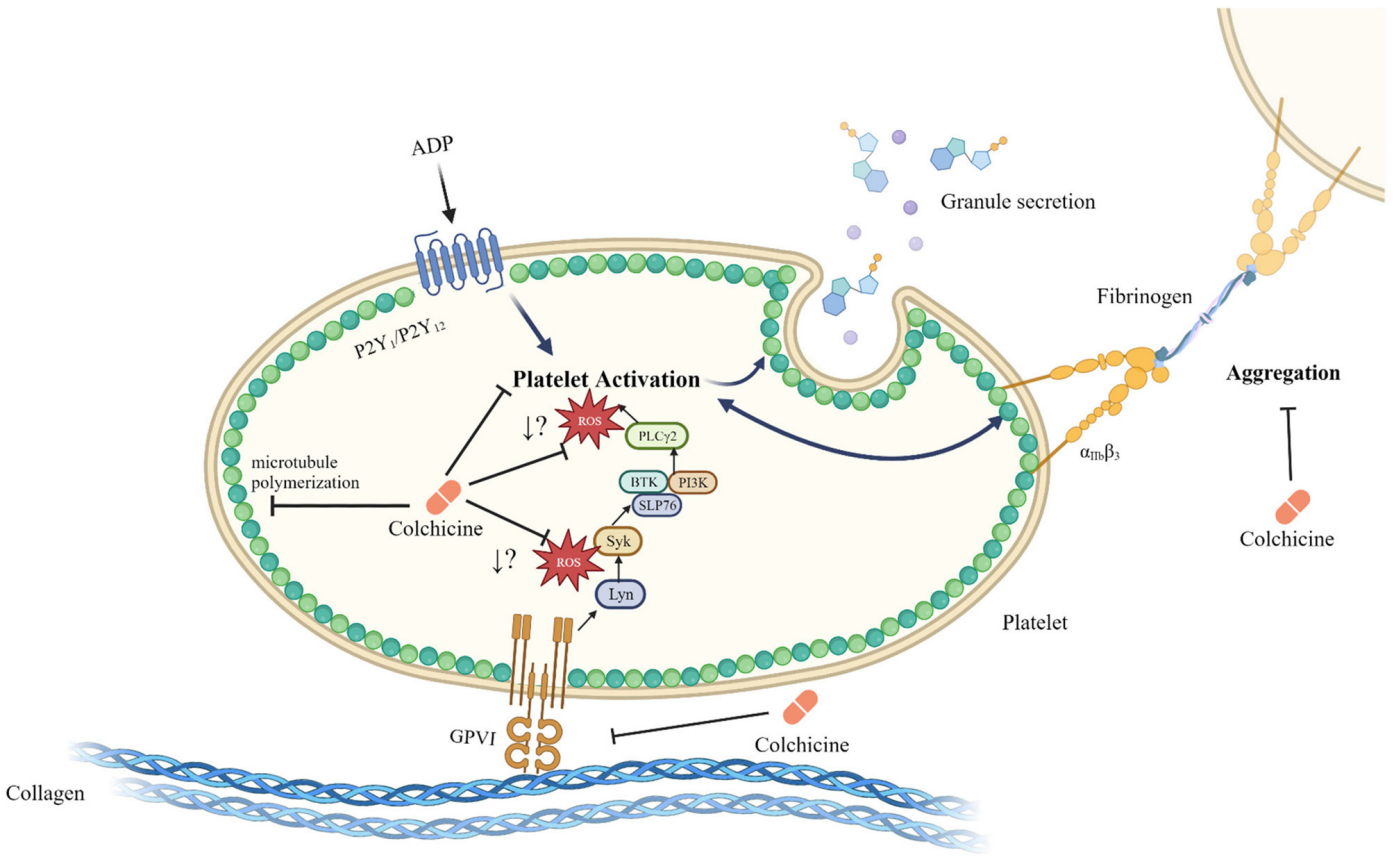
Schematic of the inhibitory role of colchicine in platelet activation. Colchicine modulates microtubule polymerization, but also leads to a reduction in platelet activation and aggregation. Colchicine does not change GPVI surface expression levels but does result in reduced ROS generation by platelets in response to stimulation of the GPVI receptor by CRP-XL; however, it is not known at what point within the GPVI signaling pathway ROS generation is affected. While the reduction in ROS generation in response to colchicine is specific to the GPVI signaling pathway, reduced activation by colchicine, particularly after stimulation with other agonists such as ADP, may be a microtubule depolymerization dependent effect. Whether colchicine directly interacts with the platelet GPVI receptor or acts exclusively downstream of this receptor is unknown. Created with Biorender.com (20, 52, 56–58).

In our studies, stimulation of GPVI with CRP-XL (cross-linked collagen related peptide, specific for GPVI stimulation) generated reactive oxygen species (ROS), and colchicine led to a reduction in ROS generation *in vitro* (20) (Figure 1). Colchicine did not cause a reduction in GPVI surface expression, indicating that the observed effect was not due to a change in GPVI receptor number but rather a change in the signaling events downstream of the receptor binding. Colchicine decreased CRP-XL-stimulated P-selectin expression and trended toward a decrease in GPIIb/IIIa conformational change. A previous study also reported on the inhibitory effects of colchicine on collagen-mediated serotonin release from platelets (59).

In our *in vitro* studies, colchicine also led to a reduction in procoagulant platelet formation in response to thrombin-collagen dual stimulation (20), and this reduction in procoagulant platelet formation could be particularly important in the setting of MI and stroke. In a sub-study of the LoDoCo2 trial, a reduction in platelet/hemostasis related markers [GPVI, proto-oncogene tyrosine-protein kinase Src (SRC) and CD40L] was noted (21) in response to colchicine lending plausibility to an *in vivo* antiplatelet effect. Given the already recognized anti-inflammatory effects of colchicine (14–18), colchicine may be more beneficial than targeting the GPVI receptor alone since it reduces both inflammatory and thrombotic responses.

Targeting the collagen receptor may be particularly relevant to at risk populations such as the elderly due to their risk of developing bleeding complications with other therapies (60). Platelets from elderly people have been shown to be hyperreactive to low dose collagen in terms of aggregation and ATP release (61). Colchicine inhibition of collagen-mediated platelet activation may therefore be particularly important in this population, especially as colchicine is not associated with increased rates of bleeding in recent cardiovascular trials. The use of colchicine in the elderly will, however, require particular care because of reduced renal function, increased susceptibility to the adverse effects of polypharmacy, and the risk of drug interactions.

## Discussion

We have outlined the importance of 1) including a broader approach to platelet function studies during clinical trials; 2) the contribution of collagen-mediated platelet activation in diseases/conditions such as CAD, MI and stroke; and 3) the potential role of colchicine in targeting collagen receptor pathways.

As current antiplatelet therapy inhibits hemostasis and can lead to bleeding complications, colchicine may represent a useful adjunctive therapy to traditional antiplatelet therapies that does not appear to increase the risk of bleeding, and may be of particular benefit in clopidogrel non-responders (62). To fully understand the mechanistic processes underlying the protective effects of any medication, and colchicine in particular, against CVD, there is a pressing need for future clinical trials to specifically evaluate collagen-mediated platelet activation. This should be done in two ways. Firstly, and most simply, studies should measure soluble GPVI which is an indicator of prior platelet receptor activation *in vivo*. Secondly, and more laboriously, platelets from patients should be stimulated *ex vivo* using collagen (or CRP-XL) to indicate the residual responsiveness of platelets to collagen. This complementary information would allow the analysis of the effect of different agents—P2Y_12_ inhibitors, antithrombins, colchicine and newer agents—on specific platelet activation pathways *via* collagen that might be more revealing than conventional agonist assays simply stimulating using ADP.

Our understanding of the effectiveness of current antiplatelet regimes would also be enhanced by broadening the usual evaluation of residual platelet activity to include other agonists. Because P2Y_12_ receptor inhibitors inhibit the secondary augmentation response, they affect more pathways than just the P2Y_12_ receptor pathway, and the inclusion of other platelet activity tests and agonists such as thrombin and collagen receptor related pathways would clarify how much residual non P2Y_12_ receptor-mediated platelet activation is evident in different clinical or therapeutic scenarios. For example, in ACS patients receiving both aspirin and clopidogrel, a loading dose of clopidogrel reduced platelet aggregation, platelet activation, and platelet-leukocyte aggregates in response to both ADP receptor and PAR-1 stimulation (63). *In vitro* inhibition with cangrelor (an intravenous reversible P2Y_12_ inhibitor) led not only to a decrease in platelet responsiveness to ADP, but also to PAR-1 and PAR-4 (thrombin receptors) agonists and CRP-XL. This diminished response was also observed in relation to the release of dense granule contents (ATP and ADP) after stimulation with PAR-1 agonist and CRP-XL (31). In addition, in this study, the VerifyNow results did not agree with the GPIIb/IIIa binding results that were established by flow cytometric analysis, and this was attributed to the restriction of the VerifyNow assay to the P2Y_12_ pathway.

Recent clinical trials targeting GPVI either directly with ACT017, Voors-Pette et al. (10) or indirectly with Revacept, Uphaus et al. (9) have shown great promise in reducing collagen-mediated platelet activation and improved stroke outcome without leading to bleeding complications (64). Colchicine reduces the risk of hemorrhagic and ischemic stroke (65–67), which is consistent with the reduction observed for Revacept both in a mouse stroke model (36) and a human carotid stenosis clinical trial (9), however colchicine has additional anti-inflammatory affects. Like ACT017 and Revacept, colchicine treatment has not been linked to bleeding complications. Other potential GPVI related targets, undergoing clinical trials and scientific investigation, have shown decreased platelet responses including aggregation, P-selectin expression and GPIIb/IIIa conformational change in response to multiple agonists, some of these targets are in use for treatment of hematological malignancies (68–70).

Although dual antiplatelet therapy has been effective in reducing cardiovascular events this has been at the risk of bleeding complications, particularly in the elderly. Colchicine and other emerging therapies may modulate platelet function in ways undetected by conventional platelet function assays. Diversifying our approach to platelet activation *in vivo*, and considering the effect of agents such as colchicine on platelets and not only inflammatory pathways may reveal new clinical opportunities for patient care without the associated bleeding risk.

## Data availability statement

The original contributions presented in the study are included in the article/supplementary material, further inquiries can be directed to the corresponding author.

## Author contributions

GP, LK, and CR wrote the first draft of the manuscript. GP, LK, CR, SG, and VC reviewed and edited the manuscript. All authors contributed to the article and approved the submitted version.
